# Buprofezin Is Metabolized by *CYP353D1v2*, a Cytochrome P450 Associated with Imidacloprid Resistance in *Laodelphax striatellus*

**DOI:** 10.3390/ijms18122564

**Published:** 2017-11-29

**Authors:** Mohammed Esmail Abdalla Elzaki, Mohammad Asaduzzaman Miah, Zhaojun Han

**Affiliations:** 1 The Key Laboratory of Monitoring and Management of Plant Diseases and Insects, Department of Entomology, College of Plant Protection, Ministry of Agriculture, Nanjing Agricultural University, Nanjing 210095, China; mamiah81@yahoo.com; 2 College of Crop Science, Fujian Agriculture and Forestry University, Fuzhou 350002, China

**Keywords:** buprofezin, Cytochrome P450, functional expression, insecticide metabolism, cross-resistance

## Abstract

*CYP353D1v2* is a cytochrome P450 related to imidacloprid resistance in *Laodelphax striatellus*. This work was conducted to examine the ability of CYP353D1v2 to metabolize other insecticides. Carbon monoxide difference spectra analysis indicates that CYP353D1v2 was successfully expressed in insect cell Sf9. The catalytic activity of CYP353D1v2 relating to degrading buprofezin, chlorpyrifos, and deltamethrin was tested by measuring substrate depletion and analyzing the formation of metabolites. The results showed the nicotinamide–adenine dinucleotide phosphate (NADPH)-dependent depletion of buprofezin (eluting at 8.7 min) and parallel formation of an unknown metabolite (eluting 9.5 min). However, CYP353D1v2 is unable to metabolize deltamethrin and chlorpyrifos. The recombinant CYP353D1v2 protein efficiently catalyzed the model substrate *p*-nitroanisole with a maximum velocity of 9.24 nmol/min/mg of protein and a Michaelis constant of Km = 6.21 µM. In addition, imidacloprid was metabolized in vitro by the recombinant CYP353D1v2 microsomes (catalytic constant Kcat) 0.064 pmol/min/pmol P450, Km = 6.41 µM. The mass spectrum of UPLC-MS analysis shows that the metabolite was a product of buprofezin, which was buprofezin sulfone. This result provided direct evidence that *L. striatellus* cytochrome P450 CYP353D1v2 is capable of metabolizing imidacloprid and buprofezin.

## 1. Introduction

Buprofezin is an insect growth regulator insecticide, mainly used to control homopteran insect pests. It is a thiadizine chitin synthesis inhibitor which kills the insect at molting by inhibiting the incorporation of *N*-acetyl-d-glucosamine (2-(acetylamino)-2-deoxy-d-glucose) into chitin. Buprofezin kills the insect by its vapour phase activity through inhalation and by direct contact through adsorption by the integument [1,2].

The small brown planthopper *Laodelphax striatellus* is an important pest of rice, maize, and wheat. Insects can be controlled using chemical insecticides; however, the long-term use of chemical control leads to the development of a serious resistance to insecticides, such as organophosphates and carbamates [3,4], neonicotinoids, pyrethroids, and insect growth regulators [5,6]. In China, imidacloprid, along with buprofezin and certain other insecticides, was recommended for controlling planthoppers when these insects developed resistance to chlorpyrifos. However, the overuse of imidacloprid and buprofezin caused insects to develop cross-resistance between neonicotinoids and insect growth regulators [7]. A great deal of research has been conducted to study the molecular mechanism of insecticide resistance in *L. striatellus*. Insecticide resistance is usually developed by two major mechanisms; metabolic resistance by enhanced detoxification enzymes and target-site insensitive mutations. Buprofezin resistance has been attributed to metabolic resistance due to the increase of the expression level of a cytochrome P450 gene in *L. striatellus* [8]. Cytochrome P450s are encoded by a superfamily of genes in different organisms. Their function is related to the metabolism of a broad range of xenobiotics, including insecticides [9]. Several P450s have been shown to be overexpressed in different resistant strains of *L. striatellus* [8,10,11,12,13].

Usually, a single P450 enzyme can metabolize only one insecticide or its homologous series. For example, QTC279 metabolizes deltamethrin in *Tribolium castaneum*, CYP6M2 metabolizes different pyrethroids in *Anopheles gambiae*, CYP337B3 metabolizes fenvalerate in *Helicoverpa armigera*, and CYP6AY1 degrades imidacloprid in *Nilaparvata lugens* [14,15,16,17]. Furthermore, few insecticide resistance-related genes have been studied for their substrate spectrum. Thus, little is known about the cross-resistance between different kinds of insecticides that is mediated by a single or multiple P450s. Therefore, our study investigates whether imidacloprid-metabolizing cytochrome P450 CYP353D1v2 is also capable of degrading the chemically unrelated insecticides buprofezin, chlorpyrifos, and deltamethrin.

## 2. Results

### 2.1. Functional Expression of CYP353D1v2 in Sf9 Cells

**Figure 1:**
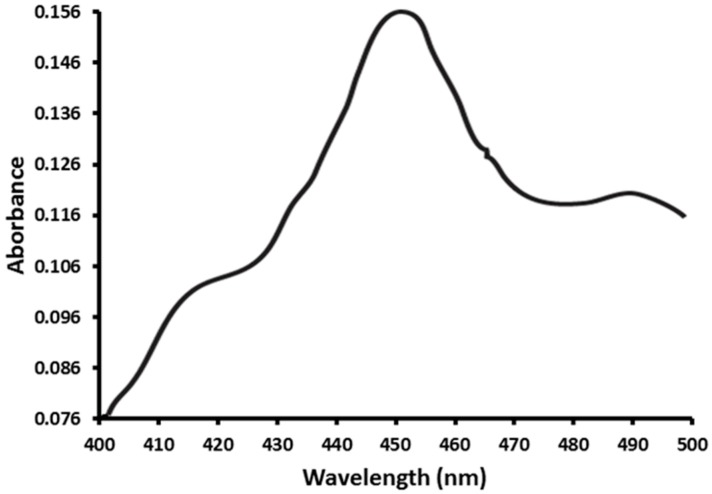
Carbon monoxide difference spectra of microsomes isolated from Sf9-cells recombinantly expressing *L. striatellus* P450, CYP353D1v2.

The CYP353D1v2 protein was expressed in insect cells using a baculovirus expression system. The reduced CO-difference spectrum showed that CYP353D1v2 was expressed predominately in its P450 form, with a distinct peak at 450 nm, and in a low level in its P420 form. This difference is indicative of a good-quality functional enzyme (Figure 1).

### 2.2. Enzyme Kinetics

**Figure 2:**
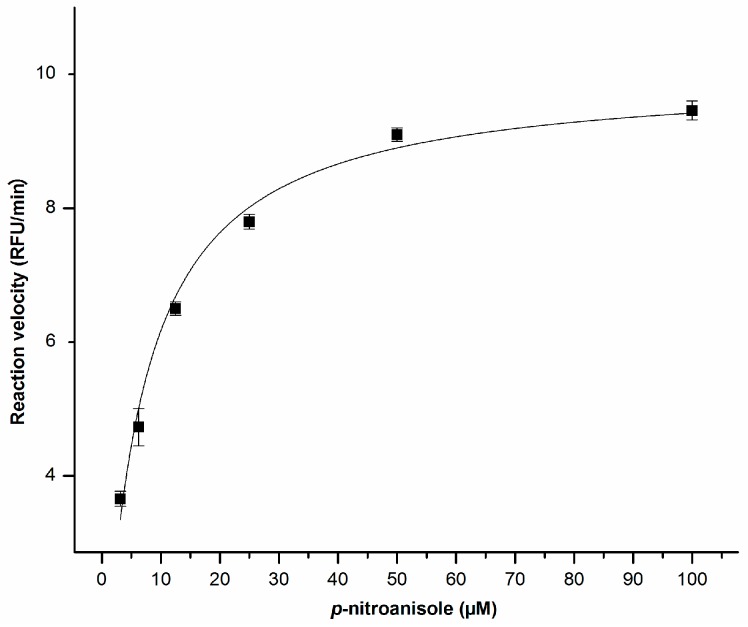
The metabolic activity of the recombinant CYP353D1v2 enzyme. Rate of dealkylation reaction of recombinant CYP353D1v2 with the fluorescent substrate *p*-nitroanisole. The Km value was determined as 6.21 ± 0.65 μM.

Our previous work revealed that the recombinant CYP353D1v2 protein efficiently catalyzed the model substrate *p*-nitroanisole with a specific activity of 32.70 pmol min^−1^ pmol^−1^ protein [18]. Thus, kinetic analysis was conducted with *p*-nitroanisole. The metabolism of *p*-nitroanisole follows Michaelis–Menten kinetics: Km 6.21 ± 0.65 µM and Kcat 0.023 ± 0.00001 µM (Figure 2).

### 2.3. Insecticides Metabolism Assay 

**Figure 3:**
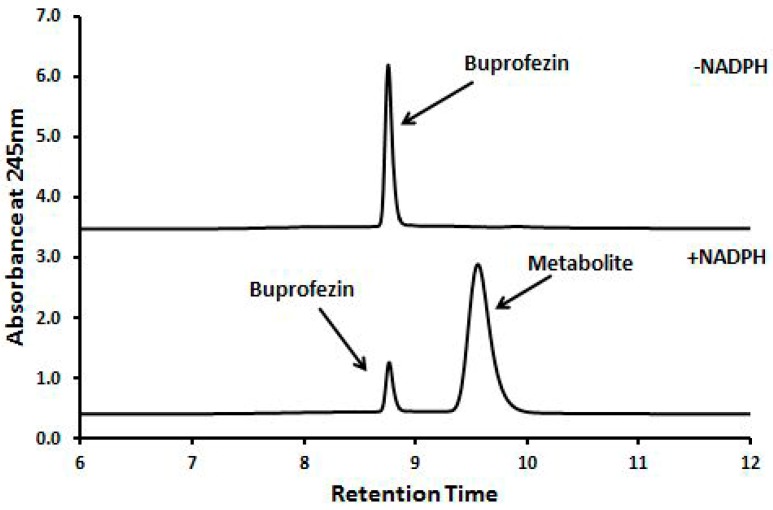
Metabolism of buprofezin by CYP353D1v2. Buprofezin depletion (eluting at 8.7 min) and metabolite formation (eluting at 9.5 min) observed after incubating the insecticide with the microsome preparation. Incubations carried out in the absence of an NADPH regenerating system showed no change when compared with the control chromatogram of the parental buprofezin.

**Figure 4:**
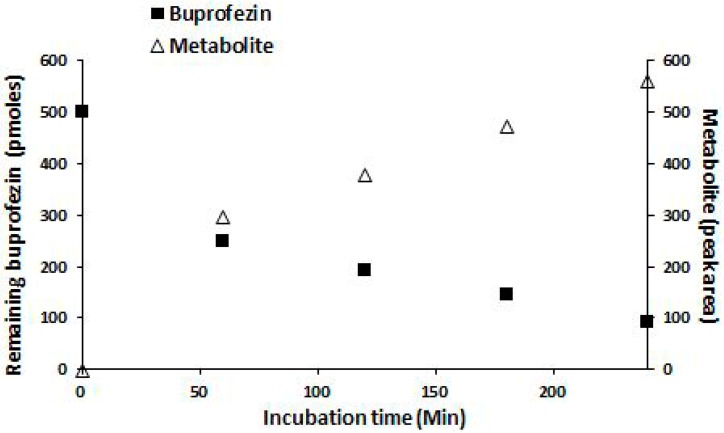
Time course of buprofezin depletion (squares) and metabolite formation (triangles). Approximately 60% of buprofezin was metabolized within 4 h. Reactions were performed at 30 °C with 100 μM buprofezin.

**Figure 5:**
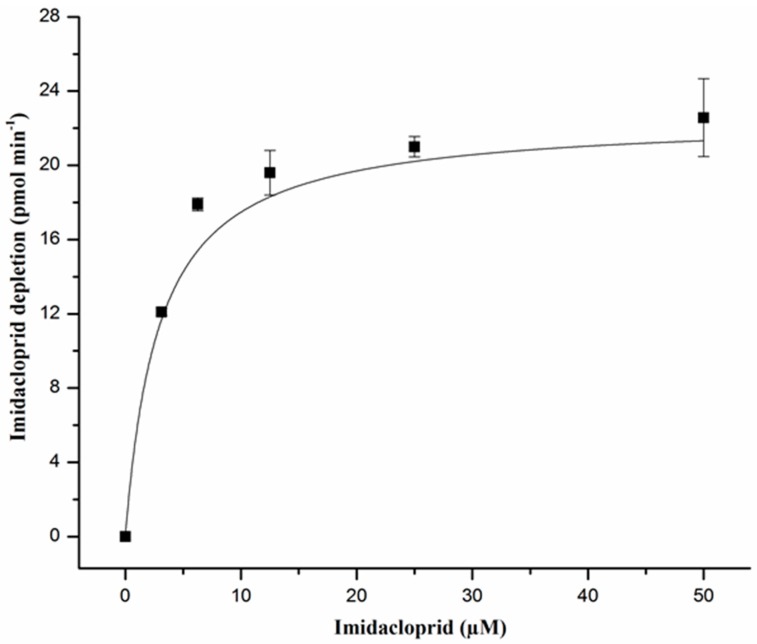
Michaelis–Menten kinetics of imidacloprid detoxification by functionally expressed CYP353D1v2, analyzed by non-linear regression. Data points are mean values ± SD (*n* = 3). The Km value was determined as 6.41 ± 1.27 μM.

The catalytic activity of CYP353D1v2 was initially assessed by measuring substrate depletion and analyzing the formation of metabolites. The NADPH-dependent depletion of buprofezin (8.7 min) and paralleled formation of an unknown metabolite (eluting at 9.5 min) was observed after incubating buprofezin with CYP353D1v2/CPR microsomes. Incubations carried out in the absence of an NADPH-regenerating system showed no change in the control chromatogram of the parental buprofezin molecules (Figure 3). Subsequently, the ability of CYP353D1v2 to metabolize buprofezin was further verified by measuring the time dependence of buprofezin depletion and metabolite formation peak (Figure 4). Incubations carried out in the absence of an NADPH-regenerating system revealed no change in the control chromatogram of the parental buprofezin molecule. The CYP353D1v2 is unable to metabolize deltamethrin and chlorpyrifos. In addition, the rate of imidacloprid depletion in response to increasing imidacloprid concentration revealed Michaelis Menten kinetics: Km 6.41 ± 1.27 μM, kcat 0.06 ± 0.0008 min^−1^, and Vmax = 24.56 pmol depleted imidacloprid min^−1^ (Figure 5). 

### 2.4. Identification of Buprofezin Metabolite

**Figure 6:**
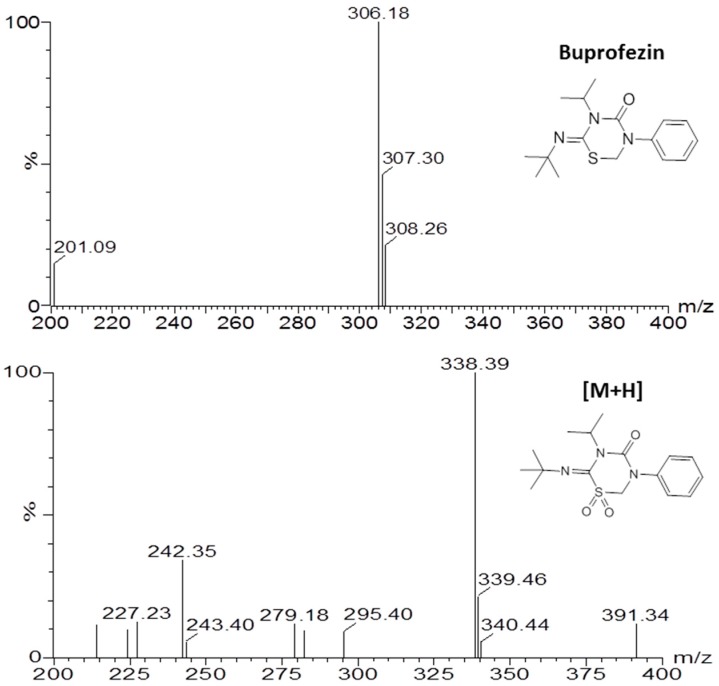
Electrospray ionization mass spectrum of the substrate buprofezin and the metabolite buprofezin sulfone. Upper panel: Mass spectra of the substrate buprofezin. Lower panel: Mass spectra of the metabolite buprofezin sulfone. Incubation of 30 pmol CYP353D1v2 and 100 μM buprofezin for 4 h.

**Figure 7:**
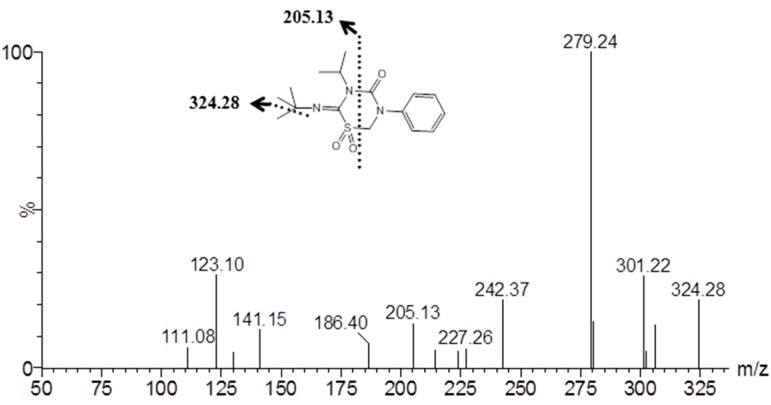
Fragment ion spectrum of the metabolite buprofezin sulfone. The dotted line and the arrow shows the fragment ions of buprofezin sulfone at [324.28]^+^ and [205.13]^+^.

Samples from the imidacloprid metabolism tests were subjected to an ultra-performance liquid chromatograph tandem mass spectrometry ultra-performance liquid chromatograph (UPLC-MS/MS). The positive ion mode mass spectrum of the major detectable metabolite was buprofezin sulfone generated from buprofezin with a molecular ion peak at *m*/*z* [M + H]^+^: 338.39 (Figure 6). The MS/MS spectrum of the metabolite showed the presence of buprofezin sulfone with several characteristic fragments at [324.28]^+^ and [205.13]^+^ (Figure 7).

## 3. Discussion

The extensive applications of buprofezin and imidacloprid resulted in cross-resistance between imidacloprid and buprofezin in *L. striatellus* [19]. Cytochrome P450 enzymes are related to insecticide resistance in different insects [20]. CYP353D1v2 has been associated to the hydroxylation of imidacloprid in *L. striatellus* [18]. However, little is known about its substrate specificity, and whether the cross-resistance among insecticides of different kinds is mediated by a single or multiple P450s in insects. Thus, this study was conducted to examine whether imidacloprid-metabolizing cytochrome P450 CYP353D1v2 is also capable of metabolizing other insecticides including buprofezin, chlorpyrifos, and deltamethrin.

Our previous study demonstrated that CYP353D1v2 expressed in Sf9 cells could degrade imidacloprid. Besides this, in the current study, insecticide degradation experiments confirmed that CYP353D1v2 is also able to catalyze the oxidation of buprofezin to buprofezin sulfone. The first evidence of a single P450 enzyme metabolizing chemically unrelated insecticide was provided when neonicotinoid-metabolizing cytochrome P450 CYP6CM1 was reported as being capable of degrading pymetrozine in *Bemisia tabaci* [21,22]. Furthermore, CYP6G1 has been proved to metabolize both dichlorodiphenyltrichloroethane (DDT) and imidacloprid in *Drosophila melanogaster* [23] and CYP6M2 to degrade both pyrethroids and organochlorine insecticide DDT in *A. gambiae* [24]. Recently, several pyrethroid-metabolizing P450s in *A. gambiae* were found to degrade a juvenile hormone analogue, pyriproxyfen [25]. Therefore, it is safe to anticipate that a single CYP enzyme could degrade different chemicals, and thus, the overexpression of some *CYP* genes in resistant insects could result in cross-resistance among chemically unrelated insecticides.

Indeed, previous studies in *L. striatellus* have reported that CYP6AY3v2 and CYP353D1v2 are associated with cross-resistance among imidacloprid, deltamethrin, and chlorpyrifos [26]. However, the present study proved that CYP353D1v2 is unable to degrade deltamethrin and chlorpyrifos, the possible reason that other P450 components may catalyze the metabolism of the parent chemicals, and CYP353D1v2 degrades the metabolite and accelerates the detoxification, such as mosquito cytochrome P450 CYP6Zs [27].

Moreover, the Michaelis constant values for imidacloprid and *p*-nitroanisole were 6.41 ± 1.27 μM and 6.21 ± 0.65 µM, respectively. The higher Km value of imidacloprid suggests that CYP353D1v2 has a lower binding affinity to imidacloprid than *p*-nitroanisole. This result calls for further research on the molecular pharmacology of buprofezin, imidacloprid, and *p*-nitroanisole metabolism.

## 4. Materials and Methods

### 4.1. Insecticide and Chemical

Buprofezin 97%, chlorpyrifos 96.5%, and deltamethrin 98% were purchased from Invitrogen Biotechnology Co., Ltd., Shanghai, China. Nicotinamide–adenine dinucleotide phosphate (NADPH), NADP+, Glucose-6-phosphate, and Glucose-6-phosphate dehydrogenase were purchased from Sigma (St Louis, MO, USA). *p*-Nitroanisole was obtained from AnaSpec INC, Fremont, CA, USA. All consumables for recombinant expression were obtained from (Invitrogen Life Technologies, Carlsbad, CA, USA).

### 4.2. Cloning and Functional Expression of CYP353D1v2 and Preparation of Microsomes

CYP353D1v2 was expressed in Sf9 cells by using a baculovirus expression system according to Elzaki et al. (2017) [18]. Briefly, full-lengths of CYP353D1v2 and cytochrome P450 reductase (CPR) coding sequences of *L. striatellus* were obtained from NCBI (GenBank access number: JX566823 and KJ017971.1, respectively). Forward and reverse primer for CYP353D1v2 was BamHI: CGCGGATCCGCCACCATGGTGAAAATAAATGGAAAG, and xho1: CGGGGTACCTTATCTATCATTGAAAAAAAT, respectively, and for CPR were BamHI: GGATCCATGGAGGTGGAGGCTGAT, and Hind111: GACAAGCTTTTAGCTCCAGACATCGGCCG. Both CYP353D1v2 and CPR were inserted into the pFastBacTM expression vector (Invitrogen Life Technologies). The constructed pFastBacTM was transformed into DH10Bac cells according to the manufacturer’s instructions (Invitrogen Life Technologies). The white colonies were used for isolating bacmid DNA according to the manufacturer’s protocol. Sf9 insect cells were transfected with recombinant bacmid DNA of CYP353D1v2 and CPR. The cells were grown to a density of 2 × 106 cells/mL and were co-infected with recombinant baculoviruses containing CYP353D1v2 and CPR with various multiplicity of infection ratios to identify optimal conditions. After further culture for 48 h, the infected cells were collected and washed with 0.1 M potassium phosphate buffer, and the microsome of the membrane fraction was prepared according to standard procedures and stored at −80 °C [28]. The protein content of the microsome samples was measured according to Bradford (1976) using bovine serum albumin as a reference [29]. P450 content was measured in reduced samples using CO-difference spectra [30]. The activity of CPR was estimated by measuring the NADPH-dependent reduction of cytochrome at 550 nm [31].

### 4.3. Enzyme Kinetics with p-Nitroanisole

Since in previous work CYP353D1v2 recorded high activity against *p*-nitroanisole [18], the kinetics parameter was assayed with 1 pmol of the recombinant enzyme with different *p*-nitroanisole concentrations (0–100 µM). As described by Elzaki et al. (2017), the reaction was initiated by the addition of 10 µL of 9.6 mM NADPH. The absorbance was read with a Spectra Max M2 reader (Molecular Devices, Sunnyvale, CA, USA) at 405 nm and 30 °C for 15 min. The Michaelis constant (Km) and maximum velocity (Vmax) were established from the plot of *p*-nitroanisole concentrations against the initial velocities through a non-linear regression, by fitting the data to the Michaelis–Menten equation using GraphPad Prism (GraphPad Software Inc., La Jolla, CA, USA) [18]. 

### 4.4. Insecticide Metabolism Assay

The metabolism of buprofezin, chlorpyrifos, and deltamethrin was assayed by incubation of the recombinant CYP353D1v2/CPR microsomes (30 pmol) in 0.1 M potassium phosphate buffer with an NADPH-regenerating system (Promega; 1.3 mM NADP+, 3.3 mM glucose-6-phosphate, 3.3 mM MgCl_2_, 0.4 U mL^−1^ glucose-6-phosphate dehydrogenase) and 100 µM insecticide at 30 °C for 4 h and a different time course. The total assay volume was 1000 µL. The corresponding microsome-insecticide incubation without the NADPH-regenerating system served as the control.

All samples had been pre-warmed for 5 min before reactions were started by the addition of the membrane preparations (CYP353D1v2/CPR microsomes). Reactions were carried out with 400 rpm shaking, were stopped at different time intervals using 100 µL of acetonitrile, and were incubated for a further 20 min to ensure that all insecticide was dissolved. The quenched reactions were centrifuged at 20,000× *g* for 10 min, and sixty microliters of the supernatant were sampled and injected at a flow rate of 1 mL/min at 30 °C for chromatogram analysis.

Buprofezin, chlorpyrifos, and deltamethrin were separated on an Acclaim C18 (5 μm, 250 mm × 4.6 mm) reverse phase analytical column and detected at 245, 290, and 230 nm, respectively. The maximum running conditions were established for different insecticides. For separating metabolites of buprofezin, time-course reactions were run with a linear gradient from 20% to 90% acetonitrile in water (*v*/*v*) over the first 4 min; followed by 90% acetonitrile for 6 min; and finally, returning to 20% acetonitrile for 4 min.

For enzyme reaction kinetics with imidacloprid, varying concentrations of imidacloprid (from 0 to 50 µM) were used. The rates of substrate turnover from three independent reactions were plotted against substrate concentration. Km, Vmax, and Kcat were determined.

### 4.5. UPLC-MS/MS Analysis

Samples were measured on an UPLC/XEVO TQ-S MS/MS system run with Electrospray Ionization (ESI) in positive-ionization mode with a capillary voltage of 5 kV and Turbo V gas temperature of 500 °C. The mass scan ranged from 100 to 500 *m*/*z*. The HPLC system was a Waters Acquity UPLC consisting of a binary solvent manager, column manager, and sample manager. The samples were run on a Waters Atlantis BEHC18 1.7 μm column (2.1 × 100 mm) running in a reversed-phase gradient mode with acetonitrile 80% as the eluent. For MS/MS analysis, the collision energy was set at 25 eV.

## 5. Conclusions

This study has proved that imidacloprid-metabolizing cytochrome P450 CYP353D1v2 is also capable of metabolizing insect growth regulator insecticide, buprofezin. However, CYP353D1v2 is unable to metabolize deltamethrin and chlorpyrifos. These findings provide a better understanding of the insecticide resistance molecular mechanism in *L. striatellus*.
